# Probing Viscoelasticity of Polymeric Coatings Using Nonlinear Dynamic Atomic Force Microscopy

**DOI:** 10.1002/smtd.202500723

**Published:** 2025-06-11

**Authors:** Lara Vivian Fricke, Nick Wansink, Michel Rosso, Urs Staufer, Pierpaolo Belardinelli, Farbod Alijani

**Affiliations:** ^1^ Department of Precision and Microsystem Engineering Delft University of Technology Delft 2628 CD The Netherlands; ^2^ AkzoNobel Rijksstraatsweg 31 Sassenheim 2171 AJ The Netherlands; ^3^ Department of Construction Civil Engineering and Architecture Polytechnic University of Marche Ancona 60131 Italy

**Keywords:** atomic force microscopy, nanomechanics, nonlinear dynamics, polymeric coatings, viscoelasticity

## Abstract

Atomic force microscopy (AFM) has evolved into a powerful instrument for examining material properties at the nanoscale. However, quantifying viscoelasticity using AFM remains a challenging task, since existing methods face sensitivity issues when it comes to the separation of viscous and elastic material properties. Here, a method is proposed that utilizes the nonlinear dynamic response of the AFM cantilever to effectively disentangle and independently quantify the dissipative and conservative parts of the tip‐sample interaction force. Through measurements on one and two component solvent‐borne coatings, it is demonstrated that the strength of the nonlinearity of AFM cantilever motion is predominantly determined by the elasticity of the sample, whereas the detuned frequency of the nonlinear resonance is contingent on the viscosity. The sensitivity of the quantified values is discussed by comparing the results to those of established multi‐frequency AFM measurements, showing good agreement. These findings underscore the effectiveness of nonlinear dynamic AFM for deciphering viscous and elastic material properties, potentially accelerating the development cycles of polymeric coating materials.

## Graphical abstract

For soft materials it is essential to characterize both the elastic and viscous material properties. Using an atomic force microscope, the hardening nonlinearity induced by a tip‐sample contact and bifurcation points can be used to separately estimate the visco‐ and elastic parts of the tip‐sample interaction force.

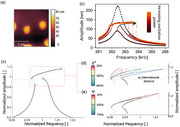

## 1 Introduction

Polymeric coatings are often utilized as one of the final protective layers in modern surface science and engineering. These coatings uniquely blend the characteristics of elastic solids and viscous fluids, exhibiting viscoelastic behavior that is crucial to their performance and durability.^[^ ^1^, ^2^ ^]^ The viscoelasticity of coatings stems from their intricate polymer microstructure that often incorporates a variety of components, including fillers and copolymers. These microstrucural components are commonly engineered to deliver coatings with tailored macroscopic mechanical behaviors.^[^ ^3^, ^4^ ^]^ Therefore, understanding the influence of micro‐ and nano‐scale viscoelasticity on the overall mechanical properties is key to designing more robust and reliable coatings.^[^ ^3^ ^]^

Conventional coatings characterization methods, such as the Dynamic Mechanical Analyzer (DMA), can determine the viscoelastic properties of the bulk material.^[^ ^5^, ^6^ ^]^ However, it fails to analyze these at the microscopic scale.^[^ ^5^ ^]^ This issue can be resolved by Atomic Force Microscopy (AFM) which can provide high‐resolution imaging, as well as analyzing nanomechanical properties of elastic materials.^[^ ^7^, ^8^, ^9^ ^]^ The determination of viscoelastic material values with AFM, however, is more complex.^[^ ^10^ ^]^ A number of methods have been developed to address this,^[^ ^10^, ^11^ ^]^ yet it remains challenging to untangle the elastic and viscous components of polymeric materials.

An AFM uses a sharp tip at the end of a cantilever to locally probe nanoscale tip‐sample forces. AFM methods can be broadly categorized into two classes: off‐resonance, also commonly known as quasi‐static techniques, and on‐resonance or dynamic AFM.^[^ ^12^, ^13^ ^]^ Off‐resonance methods, such as force‐displacement curves,^[^ ^10^, ^14^, ^15^ ^]^ or nano‐DMA,^[^ ^16^, ^17^ ^]^ directly link the deflection data of the cantilever motion with the material properties without the need for computationally intensive operations. However, they are relatively slow, and the direct contact between the AFM tip and the sample can irreversibly damage the sample. On the other hand, on‐resonance techniques mitigate the damage to the specimen, since the tip only briefly interacts with the sample.^[^ ^18^, ^19^ ^]^ To find a direct correlation between the resonant cantilever motion and the sample properties, multi‐frequency AFM methods such as bi‐modal^[^ ^20^, ^21^, ^22^ ^]^ and intermodulation AFM^[^ ^23^, ^24^, ^25^, ^26^ ^]^ are commonly used. In these methods viscoelastic properties are found via an inverse approach and by fitting the experimental observables, e.g., phase and amplitude of harmonics or resonances of the cantilever. However, this fitting process can lead to non‐unique and often non‐physical estimation of parameters,^[^ ^26^ ^]^ which introduces sensitivity issues regarding the disentanglement of viscous and elastic material properties.^[^ ^24^, ^26^ ^]^

In this paper, a method is proposed which combines the advantages of off‐resonance AFM methods, where a direct link between the experimental data and the material properties is possible, and the shorter contact times of the dynamic AFM. To characterize viscoelasticity, the proposed method leverages the nonlinear dynamic response of an AFM cantilever, owing to tip‐sample interactions. By sweeping the excitation frequency around the fundamental resonance close to the sample, we observe a pronounced nonlinear hardening response with saturated amplitude that directly correlates with the sample's elasticity. Furthermore, we note that the detuned nonlinear resonance of the cantilever due to contact is highly sensitive to dissipation, making it an effective probe for estimating the energy loss. By fitting these two distinct nonlinear features to the Derjaguin–Muller–Toporov‐Kelvin–Voigt model, we independently quantify the dissipative and conservative components of the tip‐sample interaction force, providing a direct means to obtain viscous and elastic properties. To validate the method, we further performed measurements on a well‐known reference polymer blend, as well as on solvent‐borne coatings. Additionally, we conducted intermodulation AFM (ImAFM) measurements on the same coatings, obtaining close agreement. These results demonstrate the sensitivity of the nonlinear dynamic response to viscoelastic properties, which eliminates the need for multi‐parameter fitting. As a result, the proposed method avoids the risk of non‐convex parameter estimation and ensures accurate identification of viscoelastic properties.

## 2 Results

### 2.1 Nonlinear Dynamics Identification

**Figure 1:**
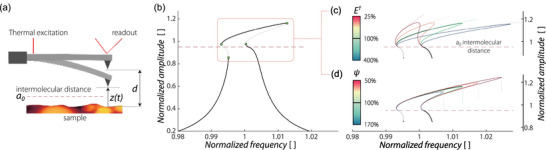
Nonlinear dynamic simulation of an AFM cantilever. a) Schematic of the experimental setup. The thermally actuated cantilever is at a distance *d* from the sample. The instantaneous tip‐sample separation, measured from the sample, is *z*(*t*). Regimes of attractive and repulsive forces swap at the intermolecular distance *a*_0_. b) Nonlinear amplitude‐frequency response curve showing the normalized amplitude of the cantilever deflection as a function of the normalized excitation frequency. The amplitude is referenced to the initial distance (i.e., 1 corresponds to touching the sample), while the excitation is normalized with respect to the fundamental frequency *f*_0_. Black and gray lines represent stable and unstable branches, respectively. The horizontal dashed line indicates the intermolecular distance *a*_0_. Dimensionless parameters used in simulation (see Equation of Motion Section) are *C*_1_ = −8 × 10^−5^, *C*_2_ = 2, *C*_4_ = 0.25, $\bar{y}=0.00505$, *B* = 1.56598, *D* = 0.0035, ${\bar{a}}_{0}=0.05$. c) Variation of the amplitude‐saturated hardening solution branch as a function of the effective tip‐sample elastic modulus $E^{✝}$. d) Variation of the amplitude‐saturated branch with respect to the nonlinear viscoelastic parameter ψ. For both (c) and (d), 100% corresponds to the reference configuration shown in panel (b).

To probe the viscoelastic properties of coatings using dynamic AFM, we model the sample as Kelvin–Voigt solid^[^ ^27^, ^28^ ^]^ and use long‐range nonlinear van der Waals (non‐contact) and Derjaguin–Muller–Toporov (contact) forces to account for tip‐sample interactions. This leads to the following expression for the force between the cantilever tip and the sample *F*_ts_

(1) $$F_{\text{ts}}\left(z\right)=\left\{\begin{array}{c} F_{\text{nc}}=-\frac{HR}{6z^{2}},\;\text{for}\;z>a_{0}\\ \;\\ F_{c}=-\frac{HR}{6{a_{0}}^{2}}+\frac{4}{3}E^{✝}\sqrt{R}{\left(\upsilon \right)}^{3/2}+\psi \sqrt{R}\sqrt{\upsilon }\;\dot{\upsilon },\;\text{for}\;z\leq a_{0}. \end{array}\right.$$

in which *z*(*t*) is the instantaneous tip‐sample separation, measured from the sample, *a*_0_ the intermolecular distance, and υ = *a*_0_ − *z* is the sample indentation (see **Figure** **1**a). Equation (1) indicates a purely attractive regime (*F*_nc_) for the cantilever (*z* > *a*_0_), where the interaction is influenced by the Hamaker constant *H* and the tip radius *R*. When *z* ⩽ *a*_0_, the regime of contact is entered (*F*_c_). Here, in addition to the adhesion $F_{\text{nc}}{|}_{z=a_{0}}$, a repulsive force proportional to the effective Young's modulus $E^{✝}$, and the square root of the tip radius is present. The effective Young's modulus is commonly known as the contact elasticity, which is composed of the elasticity of the sample and the tip.^[^ ^29^ ^]^ Since, in our case the deformation of the tip is negligible compared to the one of the sample, $E^{✝}=\frac{E_{s}}{1-\nu_{s}^{2}}$ where *E*_*s*_ and ν_*s*_ are the Young's modulus and the Poisson's ratio of the sample, respectively.

For most elastic materials, contact elasticity describes the amount of energy stored within the material. This can be compared to the elastic component of viscoelastic materials, known as the storage modulus *E*′.^[^ ^5^ ^]^ The purely elastic behavior exhibits no frequency dependence, and in our model is related to the component $\frac{4}{3}E^{✝}\sqrt{R}$. However, in contrast to purely elastic materials,^[^ ^30^ ^]^ viscoelastic materials require a model that accounts for a velocity‐dependent viscous component. This component is proportional to the nonlinear viscosity parameter ψ, capturing the dissipative nature of the material.^[^ ^27^ ^]^ The loss component for viscoelastic materials (loss modulus *E*″) stems from the friction force in Equation (1) that is $\psi \sqrt{R}\sqrt{\upsilon }\dot{\upsilon }$. The ratio of energy loss to energy storage defines the loss tangent, expressed as $\tan\delta =\frac{E^{\prime \prime }}{E^{\prime }}$ in terms of the mechanical properties of the material, and is calculated via

(2) $$\tan\delta =\frac{\left\langle F_{\text{ts}}\cdot \dot{z}\right\rangle }{\omega \langle F_{\text{ts}}\cdot z\rangle },$$

where the 〈〉 brackets represent a time average.^[^ ^31^ ^]^

For the tip‐sample interaction of Equation (1), cf.^[^ ^32^ ^]^ (see Section S3, Supporting Information), the relation of the loss tangent with the material parameters is

(3) $$\tan\delta =\frac{\omega }{2}\frac{\psi }{E^{✝}}.$$

To assess the sensitivity of the cantilever response to contact elasticity and loss tangent, we simulated the nonlinear dynamics of the AFM, conducting a parametric investigation with respect to the parameters $E^{✝}$ and ψ. To that end, we developed a model for the opto‐thermally excited cantilever dynamics under the influence of the tip‐sample force given in Equation (1) (see Figure 1a). The dimensionless equation of motion we obtained is

(4) $$\ddot{\tilde{q}}+D\dot{\tilde{q}}+\tilde{q}=-C_{1}-{\bar{F}}_{\text{ts}}\left(\bar{z}\right)+B\bar{y}\sin\left(\bar{\Omega }\tau \right),$$

in which $\tilde{q}$ is the generalized coordinate associated with the first bending mode of the cantilever (for more details see Equation of Motion Section). The equation was made dimensionless with respect to the equilibrium gap width (*d*) and the fundamental frequency of the cantilever in the absence of the tip‐sample interaction (ω_0_). The coupling between the generalized coordinate and the tip‐sample separation *z* occurs through the relation $\bar{z}=z/d=1-\tilde{q}$. The effective amplitude of the excitation generated by the opto‐thermal effect is $\bar{y}$ with dimensionless frequency $\bar{\Omega }=\Omega /\omega_{0}$. The coefficients *D*, *C*_1_, and *B* represent the modal damping, the static deflection and mode‐participation factor, respectively.^[^ ^33^ ^]^ In Equation (4) the dimensionless tip‐sample interaction force is

(5) $${\bar{F}}_{\text{ts}}\left(\bar{z}\right)=\left\{\begin{array}{c} C_{1}/{\bar{z}}^{2},\;\text{for}\;\bar{z}>{\bar{a}}_{0}\\ C_{1}/{{\bar{a}}_{0}}^{2}+C_{2}{\left({\bar{a}}_{0}-\bar{z}\right)}^{3/2}-C_{4}\sqrt{{\bar{a}}_{0}-\bar{z}}\dot{\bar{z}},\;\text{for}\;\bar{z}\leq {\bar{a}}_{0}, \end{array}\right.$$

with ${\bar{a}}_{0}=a_{0}/d$ being the dimensionless conjugate of the intermolecular distance *a*_0_. The parameters *C*_2_ and *C*_4_ depend directly on the the effective Young's modulus $E^{✝}$ and the viscosity parameter ψ, respectively (see Equation of Motion Section).

By performing numerical continuation on Equation (4), while sweeping the frequency about the fundamental resonance, one can obtain the nonlinear frequency response of the cantilever motion. In Figure 1b, we present the result of this simulation. When the cantilever approaches the surface, it first experiences the attractive force, which leads to a softening response.^[^ ^34^ ^]^ Afterwards, the probe touches the sample and the hardening nonlinearity becomes visible, thus detuning the nonlinear resonance to a higher frequency. This combined behavior leads to a *shark‐fin‐like* response of the cantilever motion that highlights the nonlinear interactions with the sample. In Figure 1b the green square dots mark the bifurcation points: i.e. points where the motion undergoes jump phenomena.^[^ ^35^ ^]^ They occur once the maximum possible frequency at a given excitation amplitude is reached. These bifurcation points exist because the stable solutions are separated by the unstable ones, where infinitesimal deviations from the ideal, unstable point rapidly drives the response toward one of the stable solutions.^[^ ^36^ ^]^

Next, it is important to determine the influence of viscoelastic parameters on the shark‐fin response. Figure 1c,d shows the effect of the elastic $E^{✝}$ and viscous ψ parameters on the frequency response. Softer samples (low stiffness) exhibit larger indentation with a reduced extension of the amplitude‐saturated hardening response (panel(c)). The effect of ψ is reflected in a shift of the bifurcation point (panel (d)). These effects are not correlated, meaning that the influence of elastic and damping parameters on the nonlinear dynamic response of the cantilever is independent and unique.

### 2.2 Experimental Measurements

**Figure 2:**
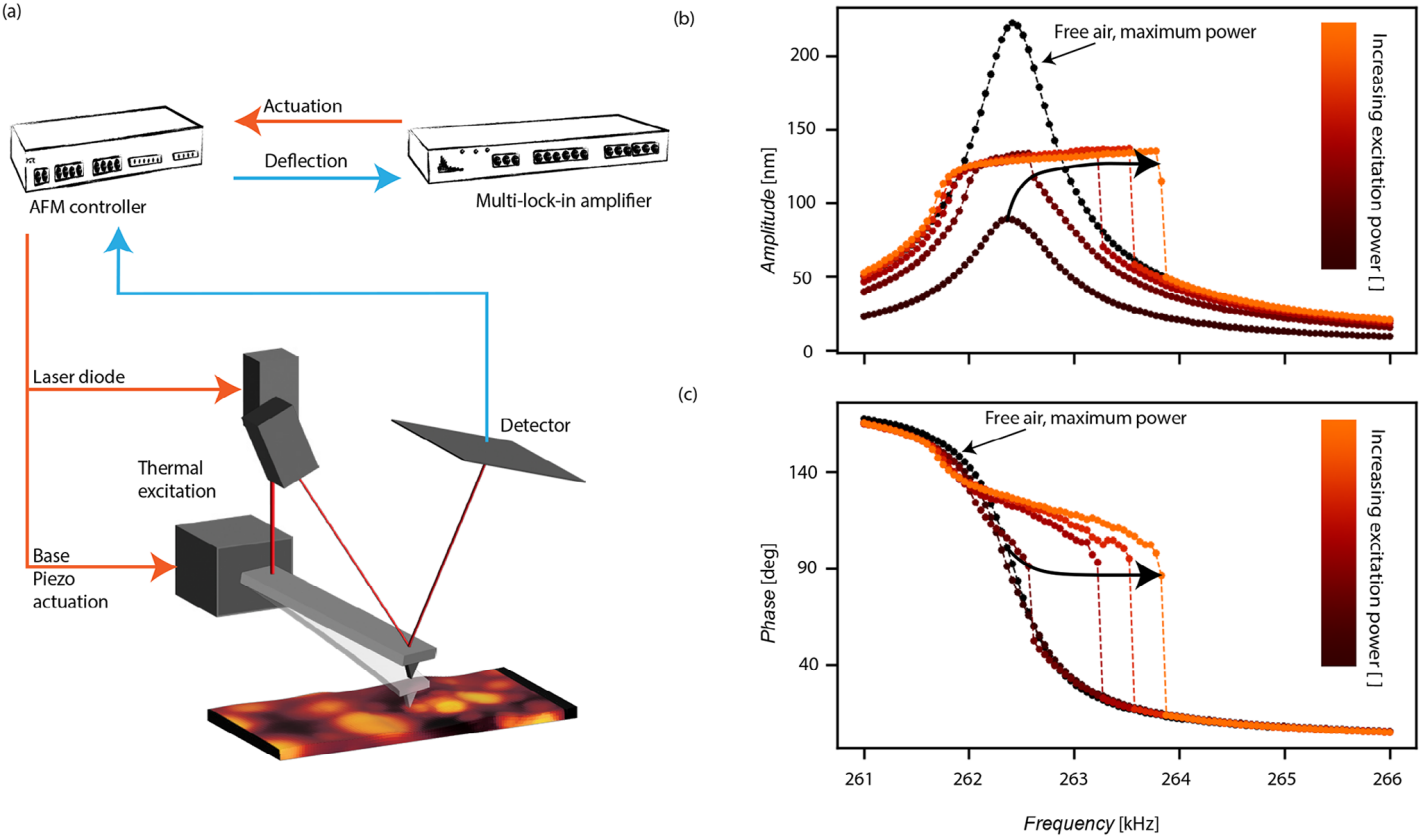
a) Experimental setup. A multi‐lock‐in amplifier is used to acquire forward and backward frequency sweep data. b) Experimentally obtained frequency forward sweep response curves using a constant cantilever‐sample distance. The color code indicates an increasing excitation power. c) Phase response of a forward sweep associated with (b). A reference measurement was performed in free air using the maximal excitation. The arrows indicate an increasing excitation force.

The measurements for the determination of viscoelastic material properties were performed using a commercial Nanosurf Drive AFM setup, see the schematic in **Figure** **2**a. The cantilever was opto‐thermally driven into resonance by means of an intensity modulated laser diode and an external multi‐frequency lock‐in analyzer, which was directly connected to the AFM control unit and controlled the magnitude of the laser excitation on the cantilever. The same lock‐in analyzer measured the output from the detector for dynamic analysis. The piezo‐scanner was set to a specific distance *d* from the surface, see also Figure 1a. To determine the exact distance *d* to the surface, a force distance curve was conducted with a certain set‐point to obtain sufficient indentation. Then, the cantilever was retracted to achieve snap‐off during every oscillation cycle. Once the force distance curve was obtained, a frequency sweep was conducted in the spectral neighborhood of the cantilever's fundamental frequency. A forward and a backward frequency sweep was performed particularly, at the same distance and with the same excitation power. Further information on the measurement setup, cantilever details and sample approach can be found in the Experimental Procedure Section below.

To analyze viscoelastic materials and fit the model of Equation 4 to experimental data, the bifurcation points resulting from forward (increase, jump down) and backward (decrease, jump up) sweeps in the frequency domain need to be found. Figure 2b,c shows a set of conducted forward frequency sweeps at a constant tip‐sample distance *d*. A full data set of experimentally obtained forward and backward frequency sweeps can be found in Section S1 (Supporting Information). Before each set of experiments, a reference measurement in free air was conducted to determine the resonance frequency of the cantilever. The excitation power was increased with every measurement also showing the multi‐stable response observed in simulations for the frequency range between the bifurcation points. However, unlike the simulations, here we notice the absence of unstable solution branches. Experimentally, these could only be obtained by implementing stabilization techniques; for example, using a closed‐loop system that allows access to otherwise unstable amplitude‐frequency data points.^[^ ^36^ ^]^ However, such methods were not applied in the present work.

Furthermore, we note that the slope of the stable branches of the amplitude until the bifurcation point is reached, stayed the same in the experiments. This aligns well with the simulations presented in Figure 1c, where the stiffness of the sample influences the slope of the frequency sweeps. Since the stiffness of the sample remained constant throughout the measurements, the slope of the amplitude while sweeping the frequency is independent of the excitation power and hence, should stay constant. This suggests that by following a reverse path from experiments, and tracking the hardening branch of the response and the associated jump frequencies (highlighted in the box region of Figure 1b), one can find the elastic and viscous properties of the sample. To that end, we developed a nonlinear identification algorithm that fitted the nonlinear dynamic responses obtained experimentally by matching the bifurcation points and the strength of nonlinearity to estimate viscoelasticity of samples.

**Figure 3:**
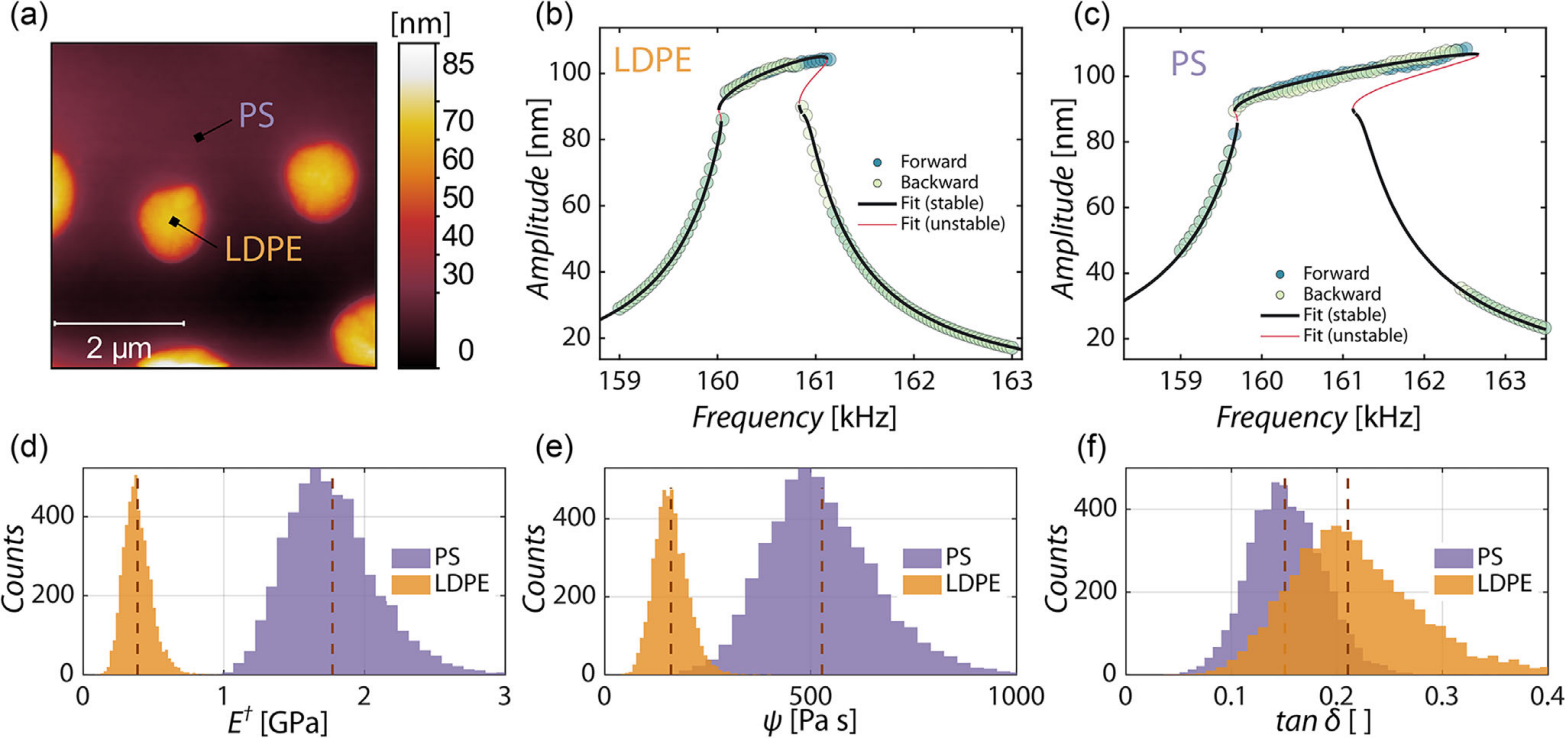
a) Topography image of PS‐LDPE blend sample obtained with a rectangular silicon cantilever Tap190GD‐G cantilever. b,c) Measurements and fits of the nonlinear frequency response curves of LDPE (b) and PS (c). Measured traces show forward and backward frequency sweeps. The solid curves show the numerical solutions for both the stable (black line) and the unstable (red line) solutions of the AFM dynamics. d–f) Histograms of the values identified from the frequency response fit (marked with a dashed line). d) Effective Young's modulus $E^{✝}$. e) Viscous coefficient ψ f) loss tangent tan δ. The histograms account for the variation of tip radius and mean distance (*d*_LPDE_=90.9 nm, *d*_PS_=91.8 nm). Dashed lines refer to the identified value for nominal *R* and *d*, i.e., $\left\{E^{✝}\;\left[\text{GPa}\right],\psi \;\left[\text{Pa s}\right],\tan\delta \right\}$={1.76, 527.83, 0.15}_PS_={0.38, 161.02, 0.21}_LDPE_.

To validate our method based on the nonlinear dynamic identification, a reference sample made of polystyrene (PS) and low density polyethylene (LDPE), purchased from Bruker Nano Inc., was tested. A topography picture of the polymer blend containing PS and LDPE is shown in **Figure** **3**a. The higher, round areas are LDPE domains which are surrounded by a homogeneous PS background. In Figure 3b,c fits of forward and backward frequency sweeps are shown for LDPE and PS, respectively. The fits are in good agreement with the experimental data. The results obtained from the fits are $E^{✝}$ = 1.76 and tan δ = 0.15 for PS and $E^{✝}$ = 0.38 and tan δ = 0.21 for LDPE, which closely match those from the literature bulk mechanical values (1.5 GPa for the storage modulus for PS^[^ ^37^ ^]^ at 30°C and 0.4 GPa for LDPE^[^ ^38^ ^]^ at 25°C). Although PS and LDPE show a large modulus difference, the algorithm developed can predict both regimes with good accuracy. For the identified ψ parameter, no equivalent value was found in the literature for PS and LDPE. The loss tangent values (0.15 for PS and 0.21 for LDPE) however, are higher compared to literature values for the bulk material (0.04 for tan δ for PS^[^ ^37^ ^]^ and 0.1 for tan δ for LDPE^[^ ^38^ ^]^ at 25°C). This makes it important to also obtain the uncertainty in the estimated values. Therefore, we performed a statistical analysis by accounting for uncertainties in both the data and the fitting process (see Figure 3d–f). These include system drift during experiment as well as the uncertainties in the tip radius and the distance from the sample. To account for the latter ones, for each parameter *p* = [*R*, *d*] samples *x*_*p*_ were generated from a Gaussian distribution with a mean µ_*p*_ and standard deviation 0.2 · µ_*p*_, i.e. ${x_{p}}_{i}∼N\left(\mu ,{\left(0.2\cdot \mu \right)}^{2}\right)$ for *i* = 1, 2, …, *N*, where *N* = 5000 is the number of samples. The generated values for *R* and *d* were employed to retrieve the parameters $E^{✝}$ and ψ from the identified *C*_2_ and *C*_4_ coefficients obtained from the fits to experimental data (see Equations (7) in Experimental Procedure Section). The distributions of material properties while accounting for uncertainties in the estimation, reveal a spread of values that does not obscure the difference between the two materials. Indeed, in both Figure 3d,e, the distributions of the properties of PS and LDPE do not overlap. The loss tangent, being a byproduct of the first two properties, suffers from cross‐uncertainties (see Figure 3f). A discussion on system drift is provided in the Section S2 (Supporting Information).

### 2.3 Characterization of Polymeric Coatings

Further, the proposed method was used to characterize two different polymeric coatings, Automotive Base Coat (ABC) and PolyUrethane Clear Coat (PU‐CC). The details of the coatings and their production processes are described in the Experimental Section.

**Figure 4:**
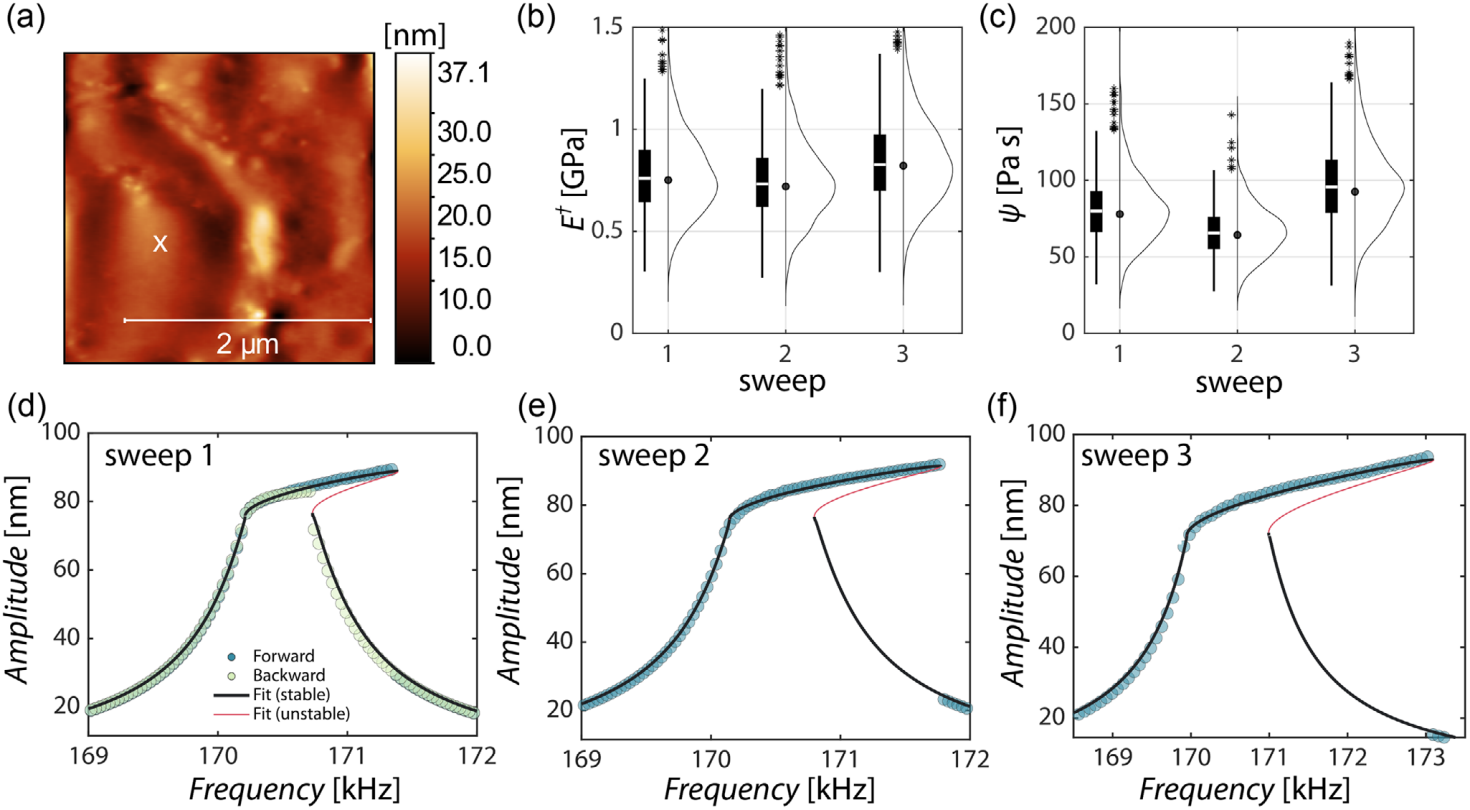
a) Topography image of ABC. b,c) Statistics for three different frequency sweeps at the same position for (b) $E^{✝}$ (nominal values 0.75, 0.72, 0.82 GPa) and (c) ψ (nominal values 77.93, 64.29, 92.54 Pa·s), these give nominal values of tan δ of 0.055, 0.047, 0.060. Initial tip‐sample distance *d*_ABC_=72.64 nm, cantilever Dyn190Al. d–f) Fit of different forward and backward frequency sweeps on the same point labeled with a cross in (a).

**Figure** **4** shows the results of the nonlinear dynamic AFM measurements on the ABC coating. In Figure 4a the topography of the coating material including the measurement position is shown. Figure 4b,c presents the fitting results for three different forward and backward frequency sweeps conducted at the same measurement position for $E^{✝}$ and ψ. The statistics for the polymeric coating are obtained similar to those of the PS‐LDPE sample in Section 2.2 by considering uncertainties in the tip‐sample distance and radius and assuming a normal distribution of their values. Before each frequency sweep, the tip was retracted and the measurement procedure followed section 2.2., which is further described in the Experimental Section. Only small deviations between the different frequency sweeps become visible, concluding that the measurement method is repeatable. Although the jump down bifurcation point of Figure 4d is at a lower frequency and a lower amplitude than those presented in (e) and (f), the fitting procedure achieves comparable fits, since the amplitude‐frequency relation can be properly identified.

**Figure 5:**
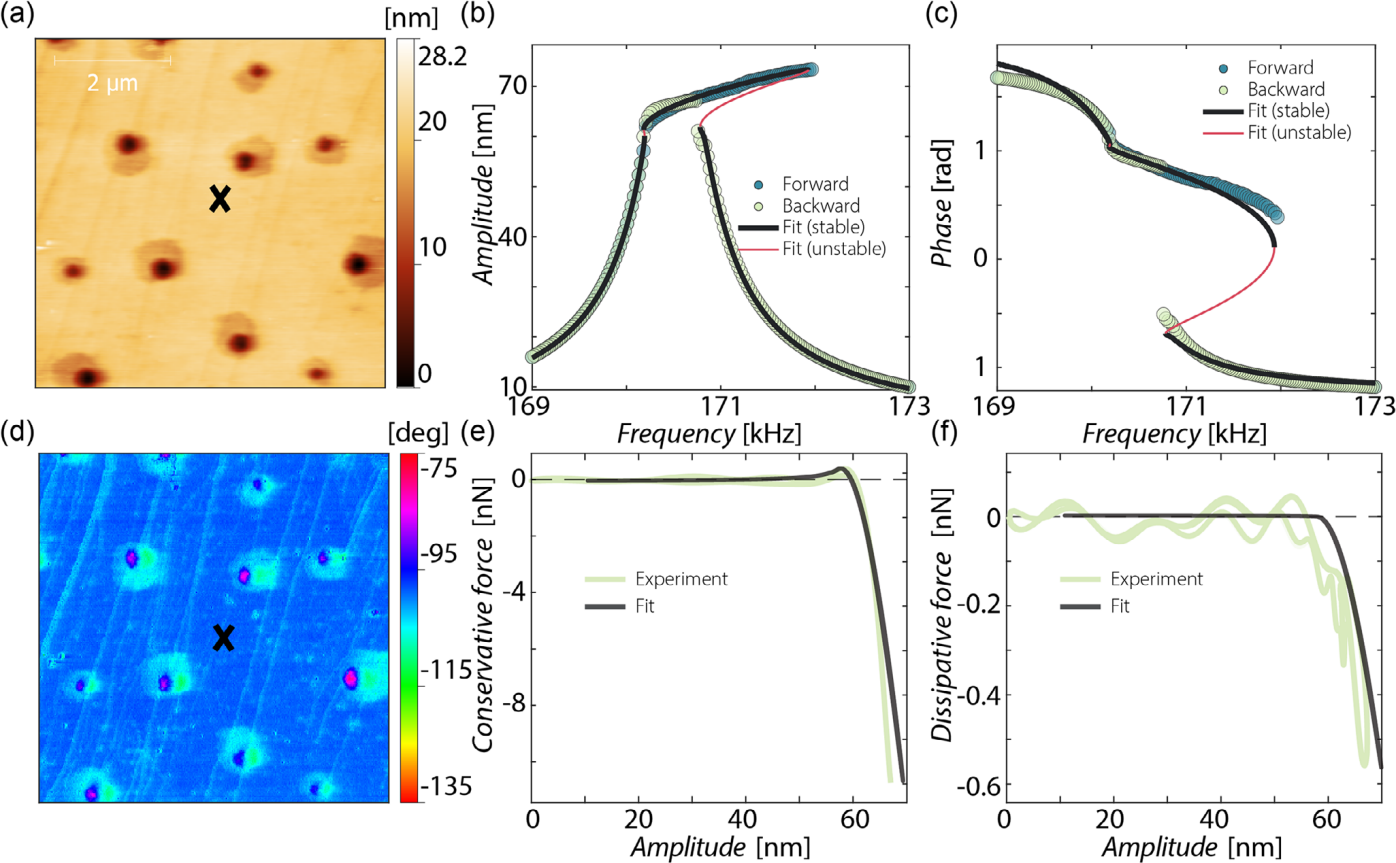
a) Topography and d) phase image of PU‐CC by ImAFM, **X** marks the measurement position. Resulting nonlinear frequency response curves for the b) amplitude and c) phase (*d*_PU‐CC_=52.52 nm, Dyn190Al cantilever). e) Conservative and f) dissipative tip‐sample forces of ImAFM on PU‐CC.

To further validate the methodology, next we compare the obtained results with those from the well‐established ImAFM technique, using the two‐component solvent‐borne coating (PU‐CC) as a test case. The details on ImAFM measurements can be found in the Experimental Section. **Figure** **5** shows the results of a study on the PU‐CC coating. The topography and phase images of the coating in Figure 5a,d, determined by ImAFM, reveal small, but noticeable dimples on a relatively smooth surface. The position for the measurements shown in (b), (c), (e), and (f) is marked with an **X** in both figures (a) and (b) respectively.

Figure 5b,c presents the results obtained by the nonlinear frequency response curves and the fits for the amplitude and phase, respectively. A good agreement between experiments and fit is observed which lead to the following identified material properties: $E_{\text{PU-CC}}^{✝}$ = 0.99 GPa, ψ_PU‐CC_ = 85 Pa·s, tan δ_PU‐CC_ = 0.045. In parallel to these measurements, we performed ImAFM measurements on the same measurement point, and obtained the conservative (Figure 5e) and dissipative (Figure 5f) components of the tip‐sample force over one oscillation cycle. This method is explained in our previous work.^[^ ^26^ ^]^ A simulation based on the model in Equation 4, adjusted for two tone excitation, and values determined by the nonlinear dynamics identification, is presented in the same figure. The results show reasonably good match between ImAFM and the nonlinear dynamic response fits for both the elastic and the viscous components, providing additional evidence on the suitability of nonlinear dynamic‐based characterization of viscoelasticity using AFM.

## 3 Discussion

As shown in earlier studies, measuring the nonlinear dynamic response enables the detection and quantification of phenomena arising from the tip‐sample interaction, such as harmonics, subharmonics, and energy transfer between modes.^[^ ^33^ ^]^ Previous efforts linking the nonlinear dynamic response with unknown parameters resulted in successful tip radius estimation^[^ ^39^ ^]^ in AFM and characterization of two‐dimensional materials.^[^ ^40^ ^]^ In this study, the nonlinear dynamic response was further leveraged to extract quantitative viscoelastic material properties, demonstrating the method's potential as a powerful tool for nanoscale mechanical characterization. An advantage of nonlinear dynamic‐based identification is its ability to independently investigate nanomechanical characteristics through specific features of the frequency response, such as hardening, softening behavior, and bifurcations. While our nonlinear resonant method is demonstrated on soft and hard polymeric coatings, it can, in principle, be applied to other softer materials such as rubbers, provided that the characteristic nonlinear dynamic features are observable in the experimental response, the cantilever‐material combination is appropriately adapted to ensure snap‐off event and suitable contact mechanics models are implemented.

The method is also less sensitive to artifacts arising from assumptions in the tip‐sample interaction force models such as the emergence of attractive forces during the retraction phase,^[^ ^28^ ^]^ as it averages over full interaction cycles and emphasizes the evolution of the response with respect to the excitation frequency.

We shall state that a number of multi‐frequency AFM methods have already been proposed for characterizing viscoelasticity, with bimodal AFM being one of the most commonly employed approaches.^[^ ^22^, ^32^ ^]^ However, unlike the method presented in this work, these techniques typically require prior knowledge of the sample's viscoelastic properties, specifically an estimated range for the loss and storage moduli, to ensure a stable and reliable fitting process. In addition, ImAFM has been successfully applied to various polymeric materials.^[^ ^24^, ^25^, ^26^, ^41^, ^42^ ^]^ While ImAFM can retrieve viscoelastic parameters, the process of obtaining the conservative and dissipative components of the tip‐sample interaction often involves non‐convex parameter estimation, which poses challenges for robust and accurate fitting.^[^ ^26^ ^]^ Nevertheless, these earlier studies have demonstrated that elastic properties of polymers can be extracted with reasonable accuracy, often showing good agreement with bulk mechanical measurements. It is also worth mentioning that compared to multi‐frequency AFM methods, the nonlinear dynamic approach presented here is relatively slow. This is largely due to the difference in data acquisition strategies: in multi‐frequency AFM, material and topographical information are acquired simultaneously in a single scan. In contrast, the nonlinear dynamic AFM methodology requires a point‐by‐point frequency sweep on a spatial grid to reconstruct viscoelastic maps which in turn increases the measurement time.

When estimating the microscopic loss tangent using the present method, we observe that the extracted values can be higher than those reported in the literature. For example, the bulk value of tan δ for PS is approximately 0.04^[^ ^38^ ^]^ at 30°C, and for LDPE it is around 0.1 at 25°C.^[^ ^38^ ^]^ which are lower than our estimations. This discrepancy may arise from the assumptions underlying our approach, which measures the loss tangent by considering harmonic indentation of the sample and fitting the jump‐down bifurcation point. Although this assumption is generally valid under soft tapping conditions, it does not account for energy leakage from the fundamental harmonic into higher‐order harmonics near the bifurcation point; an effect not accounted for in the simplified analysis used to derive Equation 2.^[^ ^33^ ^]^ Neglecting such nonlinear energy transfers can lead to the overestimation of the material's actual loss tangent, particularly in regions where nonlinear interactions can dominate the response.

From a material's characterization perspective, our AFM measurements reveal noticeable anomalies on the relatively smooth surface of PU‐CC (see Figure 5a,d). The cause of these anomalies could be *CO*_2_ gas bubbles, which can evolve during the cure of the isocyanate with hydroxyl groups; isocyanates also have a side reaction with water present in films or air, which produces *CO*_2_.

The studies performed on the micromechanical properties of coating samples using nonlinear dynamic AFM, match well the current understanding of their macroscopic behavior. We found that the effective Young's modulus of PU‐CC, see Figure 4b, is higher than that of ABC. This is in good agreement with the bulk Köning hardness measurement presented in Sample Fabrication Section, where a higher König hardness leads to a higher energy storage.^[^ ^43^ ^]^ PU‐CC is microscopically and macroscopically harder, therefore storing more energy than the polymer mixture ABC. Furthermore, our measurements show that PU‐CC is a stiff material with little dissipation ($E_{\text{PU-CC}}^{✝}$ = 0.99 GPa, ψ_PU‐CC_ = 85 Pa·s, tan δ_PU‐CC_ = 0.045). This observation has also been verified by ImAFM in Figure 5e,f. We note that the glass transition temperature of this coating material is approximately 85 °C, which was determined by DMA. The temperature during the AFM experiments in this study was 20 °C and hence, a low loss tangent and a high elasticity is to be expected.^[^ ^44^ ^]^

## 4 Conclusion

In this work, a quantitative nanomechanical method is proposed to correlate the tip‐sample interaction in an AFM with viscoelastic material properties of the sample by directly using the information of the nonlinear cantilever dynamics. The method is based on the analysis of bifurcation points observed in frequency sweeps of the cantilever, and caused by the hardening nonlinearity due to the tip‐sample interaction. It allows to effectively disentangle and independently quantify the dissipative and conservative parts of the tip‐sample interaction force compared to well‐established multi‐frequency AFM methods. The newly developed method was verified using a reference sample made of PS and LDPE. The results of the viscoelastic material properties are in good agreement with the literature values of the reference materials. The method is also capable of determining viscoelastic properties of commonly used polymeric coatings and performs well as a calibration measurement for estimating parameters using multifrequency AFM. Such quantitative estimation of viscoelasticity using the nonlinear cantilever motion could be used in future research to enhance and tailor the material properties of polymeric coatings.

## 5 Materials and Methods

### Nonlinear Frequency Response Curves

The experiments were performed using a commercial atomic force microscope (Nanosurf, DriveAFM) and two commercially available rectangular silicon cantilevers (Tap190GD‐G (Budgetsensors), Dyn190Al (Nanosurf)). The cantilevers have the same geometric dimensions. The stiffness (*k*_*Tap*190_ = 21.1 N/m, *k*_*Dyn*190_ = 27 N/m), resonance frequencies ($f_{0_{Tap190}}$ = 160.5 kHz, $f_{0_{Dyn190}}$ = 170.5 kHz), Q‐factors (*Q*_*Tap*190_ = 406, *Q*_*Dyn*190_ = 510) and tip rounding (both 10 nm according to manufacturer) are comparable. For each experiment the spring constant and resonance frequency was determined using a thermal calibration method.^[^ ^45^ ^]^ Depending on the sample, different cantilevers with different properties might be needed to achieve reproducible results. The experiments were conducted in an environmental chamber with constant temperature (20 °C) and humidity of approximately 8%. The humidity was regulated by floating the chamber with argon and maintaining a steady stream throughout the experiment. The adsorption of water on a polymer surface has been shown to influence the values of nanomechanical properties such as viscosity and the loss tangent^[^ ^32^, ^46^ ^]^ and should therefore be kept at a low and stable level. The cantilever was opto‐thermally driven by using a laser with a power of up to 10mW. The laser used for the dynamic excitation was modulated using a multilock‐in amplifier from Intermodulation Products AB.

During measurements, an unavoidable drift in the z‐position was detected. For detailed information about lateral and vertical drift see Section S2 (Supporting Information). To generate repeatable measurements and thus, minimize drift effects as good as possible, a measurement protocol was developed to obtain a proper distance *d* (see Figure 1a) to the sample. This procedure was repeated before each frequency sweep to compensate vertical drifts between measurements: (i) A force distance curve was acquired, by recording the deflection of the cantilever as function of the separation between its base and the sample. In case of low‐viscous samples, an approach set point of approximately 50 nN was found to be sufficient. (ii) The cantilever was pulled back from the sample to the desired distance *d*. This distance needed to be high enough to ensure that the cantilever can snap free in every cycle, which means that the bending force of the cantilever must be higher than the adhesive force. However, it must be close enough so that the hardening effect is observed. The true distance was determined by calculating the separation between the lowest deflection value of the force distance curve (*a*_0_) and the ending point of the retraction curve. The true distance between the tip and the sample is stated in each figure caption for the corresponding measurements. The applied oscillation amplitude and distance result in an indentation depth of approx. 20 nm in all of our experiments. iii) While maintaining this static position (*d*), the drive frequency was swept around the resonance frequency of the cantilever.

### Intermodulation AFM Measurements

In ImAFM the cantilever is excited by two tones equally spaced around its fundamental resonance frequency. The interaction of the cantilever with the sample, generates intermodulation products (IMP's), i.e., frequency peaks with a distance of Δ*f* between them. In our case, 32 amplitudes and 32 phase components of these intermodulation peaks have been recorded on a PU‐CC sample as experimental input for the viscoelastic identification procedure.

The ImAFM experiments were performed using the same commercial atomic force microscope (Nanosurf, DriveAFM) combined with a multi lock‐in amplifier from Intermodulation Products AB. The multi lock‐in amplifier was used to measure, control and analyze the frequency components resulting from the tip‐sample interaction. All measurements were conducted using a rectangular silicon cantilever Dyn190Al (Nanosurf). The stiffness of the cantilever (*k* = 19.2 N/m), its resonance frequency (*f*_0_ = 170.6 kHz) and the quality factor (*Q* = 368) were determined using a thermal calibration method.^[^ ^45^ ^]^ The cantilever was driven by two tones with Δ*f* = 464 Hz (equal to the mechanical bandwidth) equally spaced around the resonance frequency using an intensity modulated laser diode. This ensures sufficient IMP's in the frequency band to provide information about the tip‐sample interaction, while maintaining a reasonable scanning speed.^[^ ^23^ ^]^

When exciting the cantilever with two equidistant tones from resonance the amplitude is modulated in time by the frequency spacing Δ*f*, thus, forming a slowly evolving amplitude envelope. Due to the tip‐sample interaction, the amplitude of the cantilever motion increases and abruptly interacts with the surface at a certain amplitude based on the viscoelastic properties of the sample surface and the system parameters.

To extract the viscoelastic properties, the tip‐sample force is analyzed based on its in‐phase and out‐of‐phase components, knowing the amplitude and phase of IMPs and following the procedure detailed out in our earlier work.^[^ ^26^ ^]^ These components of the tip‐sample force, are only affected by the respective conservative and dissipative parts of the interaction.^[^ ^47^ ^]^ Figure 5e displays the conservative force, or virial. Similar to force‐distance curves, an attractive force close to the surface, is followed by a sharp onset of repulsive interaction at the moment of mechanical contact with the surface. On softer materials or using low stiffness cantilevers, the conservative force stays in the net attractive regime.^[^ ^48^ ^]^ The dissipative force quadrature shown in Figure 5f, increases significantly in the repulsive region.

### Equation of Motion

We determined the dynamical equation for the AFM, initially resting in static equilibrium at a distance *d* from the sample, as shown in Figure 1a. The mathematical framework to develop the continuous model for the AFM cantilever motion is within the Euler–Bernoulli assumptions. The AFM cantilever has a length *L*, mass density ρ, Young's modulus *E*, area moment of inertia *I*, and cross‐sectional area *A*. The beam is clamped at *x* = 0 and free at *x* = *L*. The dynamic deflection of the cantilever *u*(*x*, *t*) about the static deflection w*(*x*) is governed by the equation 

(6) $$\rho A\ddot{u}\left(x,t\right)+\mathrm{EI}\left(u^{\prime \prime \prime \prime }\left(x,t\right)+w^{*\prime \prime \prime \prime }\left(x\right)\right)=F_{\text{ts}}\left(z\left(t\right)\right)\delta \left(x-L\right)+\mathrm{Ysin}\left(\Omega t\right)$$

The equation was modified with respect to those presented in^[^ ^49^, ^50^ ^]^ to address the presence of opto‐thermal excitation. Surface effects related to the driving laser, which induces thermal stress (cf.^[^ ^51^, ^52^ ^]^), were modeled as an effective direct drive. The interaction with the sample is described by the force *F*_ts_ of Equation (1). The force, function of the tip‐sample separation *z* = *d* − *u*(*L*, *t*), was applied at the free end of the cantilever (δ is the dirac's delta). Equation (6) was discretized through a projection by utilizing the Galerkin spectral reduction. The analysis was limited to a single degree of freedom *q* with mode shape ϕ(*x*), that is *u*(*x*, *t*) = ϕ(*x*)*q*(*t*). This results in Equations (4) and (5) with

(7) $$\begin{array}{ccc} C_{1} & = & -\frac{HR\phi {\left(L\right)}^{2}}{6\rho Ad^{3}{\omega_{0}}^{2}\int_{0}^{L}\phi^{2}\left(x\right)dx},\;C_{2}=\frac{4E^{✝}\sqrt{R}\sqrt{d}\phi {\left(L\right)}^{2}}{3\rho A{\omega_{0}}^{2}\int_{0}^{L}\phi^{2}\left(x\right)dx},\\ C_{4} & = & \frac{\psi \sqrt{R}\sqrt{d}\phi {\left(L\right)}^{2}}{\rho A\omega_{0}\int_{0}^{L}\phi^{2}\left(x\right)dx},\;B=\frac{\phi \left(L\right)\int_{0}^{L}\phi \left(x\right)dx}{\int_{0}^{L}\phi^{2}\left(x\right)dx}. \end{array}$$

Due to the non‐smooth nature of the tip‐sample force, the numerical implementation requires re‐arrangement of the dynamics in a hybrid system formulation.^[^ ^50^ ^]^ To that end, we performed numerical continuation along a family of multi‐segment periodic orbits by making use of the HSPO functions of the Computational Continuation Core toolbox (COCO).^[^ ^53^ ^]^

### Sample Fabrication

Two different kinds of coatings were produced for this study. The first coating, presented in Figure 4, is a solvent‐borne one component coating. This means the polymer blend is fully dissolved in organic solvents. In the present study, the Automotive Base Coat (ABC) refers to a simplified variation of a commercial coating, Sikkens AutoBase Plus, frequently used as solvent‐based basecoat for metallic, pearl and solid finishes in automotive applications. This type of coating was prepared as a simple blend of commercial polymer solutions and some additional butyl acetate to reduce viscosity. For simplicity, wetting, flow or rheology additives were omitted from the formulation. The blend consisted of a hard resin (König hardness of 121 ± 1 osc.), a softer thermoplastic acrylic copolymer (König hardness of 83 ± 1 osc.) and a very soft polyester binder (König hardness ⩽ 10 osc.). Coatings were applied with a wire bar applicator to obtain dry film thicknesses of 40 ± 1 µm, and were left to dry for at least two days at 50 °C, after which the films have reached their maximum König hardness. The dry layer thickness was determined on aluminium panels with a Fisher impedance probe DualScope MP0R‐FP. The overall König hardness of the ABC coating amounts to 61 oscillations.

The second coating presented in Figure 5 is made of polyurethane clearcoat (PU‐CC). PU is commonly used as clearcoat in many applications, e.g. aerospace industry. As an example of this two component PU‐CC coating, the solvent‐based clear coat Aviox CC UVR (AkzoNobel) was investigated. For the coating preparation, component A (polyol resin solution in organic solvent) was mixed with component B (solution of isocyanate‐based hardener) in organic solvents and directly sprayed onto the substrates to a dry layer thickness of about 27 ± 1 µm. The substrates were left to cure for at least two weeks at room temperature before further testing. For this coating type, the overall König hardness amounts to 134 oscillations.

For the König hardness measurements, coatings were applied on aluminum panels and allowed to cure for two weeks. After curing, they were measured with an automatic pendulum hardness tester (Byk Gardner) at a room temperature of 21 °C. Values are reported as described in the ASTM standard.^[^ ^43^ ^]^

## Conflict of Interest

Employment: M.R. AkzoNobel. The authors declare no further competing interests.

## Author Contributions

L.‐V.‐F. and N.‐W. contributed equally to this work. P.B. and F.A. conceived the idea. M.R. fabricated the coatings. L.F., and N.W. collected the data and performed the experiments. P.B. formulated the theoretical modeling, and performed the fitting with the experimental data. N.W. and P.B. conducted analytical analysis, L.F., N.W., and F.A. designed the experiments. The project was supervised by F.A, P.B., and U.S. All authors contributed to the data analysis, interpretation of the results, writing of the manuscript.

## Supporting information

Supporting Information

## Data Availability

The data that support the findings of this study are available from the corresponding author upon reasonable request.
