# Supplementary material for: Probing Viscoelasticity of Polymeric Coatings Using Nonlinear Dynamic Atomic Force Microscopy
Source: Small Methods. 2025 Jun 11;10(2):2500723. doi: 10.1002/smtd.202500723 (PMC12825344; doi:10.1002/smtd.202500723)
Supplement: Supplementary file 1 — Supporting Information [file SMTD-10-2500723-s001.pdf]

Supplementary Information: Probing Viscoelasticity of Polymeric Coatings Using Nonlinear Dynamic Atomic Force Microscopy

Lara Vivian Fricke[†] [*], *Nick Wansink*[†], *Michel Rosso*, *Urs Staufer*, *Pierpaolo Belardinelli*, *Farbod Alijani*

[†]These authors contributed equally to this publication.
L. V. Fricke (l.v.fricke@tudelft.nl), N. Wansink (n.wansink@tudelft.nl), U. Staufer (u.staufer@tudelft.nl), F. Alijani (f.alijani@tudelft.nl)
Department of Precision and Microsystem Engineering, Delft University of Technology, Delft 2628 CD, The Netherlands
*l.v.fricke@tudelft.nl
M. Rosso (michel.rosso@akzonobel.com)
AkzoNobel, Rijksstraatsweg 31, 2171 AJ Sassenheim, The Netherlands
P. Belardinelli (p.belardinelli@staff.univpm.it)
Department of Construction, Civil Engineering and Architecture, Polytechnic University of Marche, Ancona, Italy

S1 Forward and backward frequency sweeps

In Figure 2 of the main text only forward sweeps, in amplitude and phase values, are presented. To show a complete set of experimental data for the amplitude, in Figure S1 frequency sweeps in the forward direction (panel (a)) and backward direction (panel (b)) are presented. The measurements were performed on the same PS and LDPE sample shown in Figure 3 in the main text. A B50-NCH (Nanotools) cantilever was used, which was also used for the results presented in Figure 2 in the main paper. The stiffness was $k_{B50-NCH}$=19.8 N/m, resonance frequency was $f_{0_{B50-NCH}}$=262.5 kHz, Q-factor was $Q_{B50-NCH}$=453 and tip rounding amounted to 50 nm, according to manufacturer. The experiments were performed on PS. The measurement procedure was as described in "Materials & Methods 5.1". However, a different measurement position on the sample was chosen compared to Figure 2 in the main paper. The distance d in Figure 2 in the main text amounted to 36 nm and here d was 35 nm. This is why the forward sweeps look different in the main text compared to here. Before each set of experiments, a reference measurement was conducted in free air to determine the resonance frequency of the cantilever. For the frequency forward sweeps the amplitude response leans over to the right side and for the backward sweeps it leans over to the left side compared to the resonance frequency in free air. For the fits presented in Figure 3, 4 and 5 in the main text as well for Figure 4 in the SI, always backward and forward sweeps were analyzed.

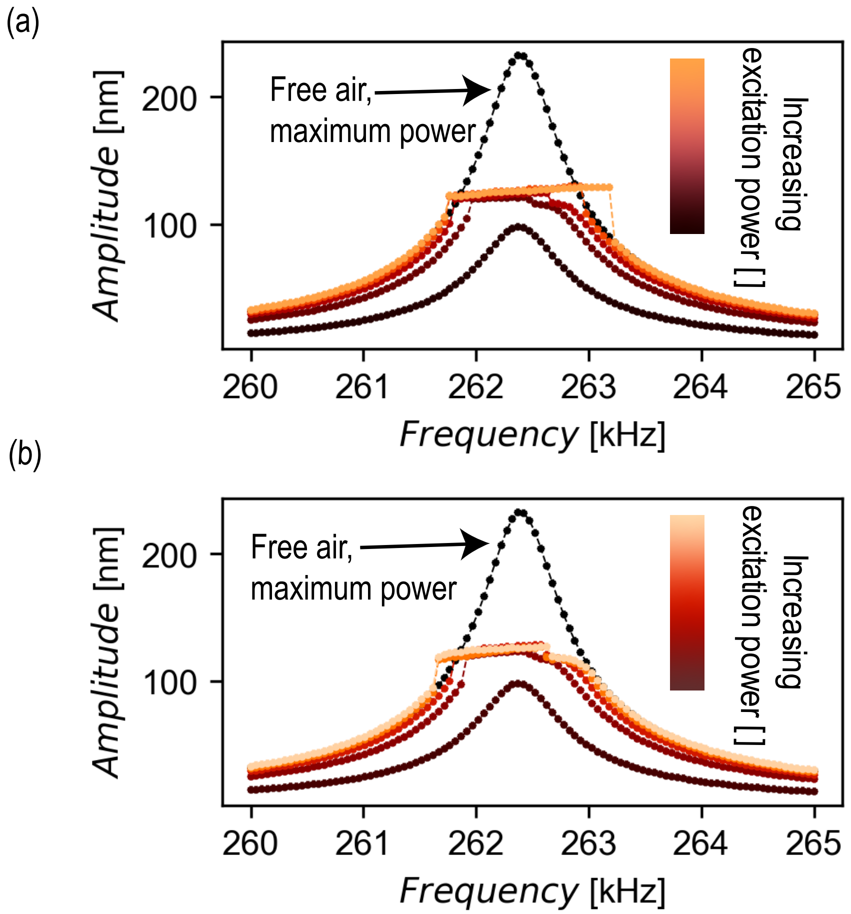

Figure S1: Experimentally obtained frequency forward sweep response curves using a constant cantilever-sample distance. The color code indicates an increasing excitation power. A reference measurement was performed in free air using the maximum excitation. (a) Forward sweeps, (b) backward sweeps.

S2 Drift in nonlinear dynamic AFM measurements

Fluctuations in both the actuation and read-out laser could lead to uncertainty in the measurements and fitting process, manifesting themselves as a drift in the frequency and amplitude response. Additionally, the distance d was not perfectly stable while conducting the frequency sweep due to drift in the z-piezo, which is responsible for the height positioning.

We investigated the effect of drift by repeating measurements in a sequence to observe changes in the dynamical response. Consecutive forward sweeps were performed at the same location on the sample. Figure S2 (left) shows the effect of the frequency drift. This affects the precise identification of the saddle-node bifurcation point, where the cantilever undergoes a jump-down during the forward frequency sweep. The amplitude drift introduces uncertainty in estimating the sample penetration along the amplitude-saturated branch (Figure S2 (right)). This strongly suggests the need to develop a rapid procedure that minimizes the influence of drift on the frequency response, thereby improving the identification of elastic and viscoelastic properties. In order to minimize the influence of the system drift, as described in "Material & Methods 5.1" in the main paper, before each frequency sweep the cantilever was retracted and approached again so that the z-piezo drift was as minimal as possible.

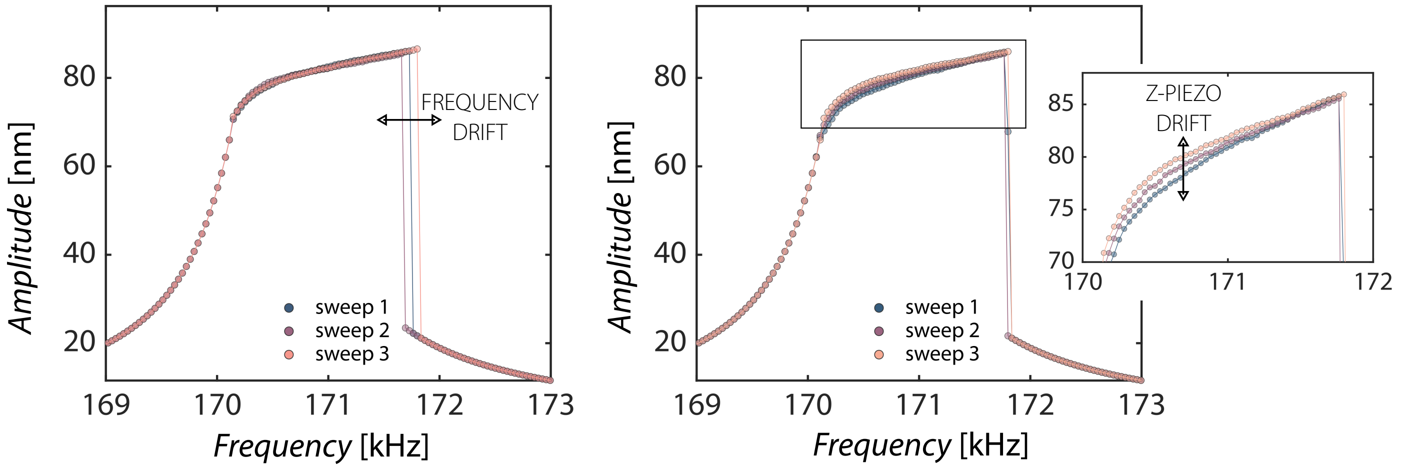

Figure S2: Frequency response illustrating drift over consecutive measurement repetitions at a fixed distance and excitation amplitude. The left panel shows frequency drift, while the right panel displays amplitude variations. Measurements are conducted on the same ABC coating sample as in the paper (Figure 4 (a) of the paper).

S3 Relation between the loss tangent and material parameters

The viscoelastic loss tangent (Equation 2 of the main manuscript) measures the ratio of energy dissipated to energy stored in one cycle of a periodic deformation of the sample for a cantilever with resonance frequency f_1. To calculate the energy stored we use the virial definition

$$V_1 = \langle F_{\mathrm{ts}} \cdot z \rangle = f_1 \int_0^{1/f_1} F_{\mathrm{ts}}(t) z(t) dt. \tag{S1}$$

The above general expression has been solved analytically for a viscoelastic force in Benaglia et al.[1] i.e.

$$V_1 = -\frac{1}{\pi} \int_0^{\upsilon_{max}} k_{\mathrm{ts}}(\upsilon) \sqrt{2A_1} \sqrt{\upsilon_{max} - \upsilon}\; d\upsilon \tag{S2}$$

in which A_1 and υ_{max} are the oscillation amplitude and the maximum indentation, respectively. Furthermore, k_{ts} is the interaction stiffness coefficient related to the velocity-independent part of F_c (Equation 1 of the main manuscript). This reads

$$k_{\mathrm{ts}}(\upsilon) = \frac{dF_{\mathrm{ts}}}{d\upsilon} = 2E^{\dagger} \sqrt{R} \sqrt{\upsilon}. \tag{S3}$$

Thus, the solution of Equation (S2) with (S3) is

$$V_1 = -\frac{E^{\dagger}}{4} \sqrt{2RA_1} {\upsilon_{max}}^2. \tag{S4}$$

Similarly, for the energy dissipated we have

$$E_{\mathrm{dis}} = \frac{\langle F_{\mathrm{ts}} \cdot \dot{z} \rangle}{\omega} = \frac{1}{2\pi} \int_0^{1/f_1} F_{\mathrm{ts}}(t) \dot{z}(t) dt. \tag{S5}$$

In the special case of a viscoelastic-like tip-sample interaction, it yields

$$E_{\mathrm{dis}} = -\frac{1}{\pi} \int_0^{\upsilon_{max}} g_{\mathrm{ts}}(\upsilon) \sqrt{2A_1} \sqrt{\upsilon_{max} - \upsilon}\; d\upsilon \tag{S6}$$

where $g_{\text{ts}} = \psi\omega\sqrt{R}\sqrt{\upsilon}$ is the effective damping coefficient.[1] The solution of Equation (S6) provides

$$E_{\text{dis}} = -\frac{\omega\psi}{8}\sqrt{2RA_1}\upsilon_{max}{}^2. \tag{S7}$$

Finally, the quantity $\frac{E_{\text{dis}}}{V_1}$ is the loss tangent

$$\tan\delta = \frac{\omega}{2}\frac{\psi}{E^{\dagger}}. \tag{S8}$$

References

[1] S. Benaglia, C. A. Amo, and R. Garcia, "Fast, quantitative and high resolution mapping of viscoelastic properties with bimodal afm," *Nanoscale*, vol. 11, pp. 15289–15297, 2019.
